# Membrane Efflux Pumps of Pathogenic *Vibrio* Species: Role in Antimicrobial Resistance and Virulence

**DOI:** 10.3390/microorganisms10020382

**Published:** 2022-02-07

**Authors:** Jerusha Stephen, Manjusha Lekshmi, Parvathi Ammini, Sanath H. Kumar, Manuel F. Varela

**Affiliations:** 1 QC Laboratory, Harvest and Post-Harvest Technology Division, Central Institute of Fisheries Education (CIFE), Seven Bungalows, Versova, Andheri (W), Mumbai 400061, India; jeruselva@gmail.com (J.S.); manjusha@cife.edu.in (M.L.); sanathkumar@cife.edu.in (S.H.K.); 2 Department of Biotechnology, Cochin University of Science and Technology, Kochi 682022, India; parubfsc@gmail.com; 3 Department of Biology, Eastern New Mexico University, Portales, NM 88130, USA

**Keywords:** bacteria, antimicrobial resistance, multidrug resistance, cholera, multidrug efflux pump, infection

## Abstract

Infectious diseases caused by bacterial species of the *Vibrio* genus have had considerable significance upon human health for centuries. *V. cholerae* is the causative microbial agent of cholera, a severe ailment characterized by profuse watery diarrhea, a condition associated with epidemics, and seven great historical pandemics. *V. parahaemolyticus* causes wound infection and watery diarrhea, while *V. vulnificus* can cause wound infections and septicemia. Species of the *Vibrio* genus with resistance to multiple antimicrobials have been a significant health concern for several decades. Mechanisms of antimicrobial resistance machinery in *Vibrio* spp. include biofilm formation, drug inactivation, target protection, antimicrobial permeability reduction, and active antimicrobial efflux. Integral membrane-bound active antimicrobial efflux pump systems include primary and secondary transporters, members of which belong to closely related protein superfamilies. The RND (resistance-nodulation-division) pumps, the MFS (major facilitator superfamily) transporters, and the ABC superfamily of efflux pumps constitute significant drug transporters for investigation. In this review, we explore these antimicrobial transport systems in the context of *Vibrio* spp. pathogenesis and virulence.

## 1. *Vibrio* Species of Human Health Significance

The *Vibrio* genus (Class Gammaproteobacteria, Family Vibrionaceae) is one of the most important food pathogens of human health significance in seafood. Vibrios are naturally present in freshwater, marine, and estuarine environments and are found in variable numbers in all kinds of seafood [1]. Their numbers can differ depending on several factors such as the season and physical parameters of water, such as temperature, salinity, and nutrient concentrations. Only a dozen among 100 named *Vibrio* species have been isolated from humans [2], representing a growing health threat to humankind. Infections due to *Vibrio* spp. usually ensue from exposure to contaminated water or consumption of raw or undercooked contaminated fish and shellfish [3]. However, person-to-person transmission has also been documented in *Vibrio* infections [1]. *Vibrio* infections in humans can be categorized into two groups: cholera and non-cholera infections. *Vibrio cholerae* causes a severe diarrheal illness, cholera, through ingestion of contaminated food or water. Non-cholera *Vibrio* species such as *V. parahaemolyticus* and *V. vulnificus* are widely distributed in coastal-marine waters. They can cause various infections such as gastroenteritis, wound infections, septicemia, etc., and are responsible for the majority of seafood-borne infections worldwide [4]. Cholera continues to be a gripping problem in developing countries like Asia, Africa, and Latin America but is rare in the developed world. Contrastingly, *V. parahaemolyticus* and *V. vulnificus* outbreaks are also common in developed countries.

### 1.1. Vibrio cholerae

The *V. cholerae* bacterium is the causative agent of the global disease cholera, with an estimated 1.3–4 million cases and 21,000–143,000 deaths worldwide every year [5]. *V. cholerae* is associated with chitinous organisms such as copepods, crustaceans, and microalgae, like zooplankton and fish [6]. *V. cholerae* bacteria are classified broadly as O1 and non-O1 serovars based on their observative agglutination in the presence of O1 antiserum. O1 *V. cholerae* and a non-O1 *V. cholerae* (O139 Bengal) have the potential to cause epidemic or pandemic cholera, and all other non-O1 and non-O139 *V. cholerae* do not infect humans or only cause mild illnesses [7]. The virulence of *V. cholerae* O1 and O139 Bengal is primarily due to their ability to produce cholera toxin CTX and cause epidemic diarrhea [7]. These two pandemic strains exist as natural inhabitants of aquatic ecosystems, making them facultative human pathogens. *V. cholerae* O1 serogroup has two biotypes (classical and El Tor) and three serotypes (Ogawa, Inaba, and Hikojima). Amongst them, the highly rampant serotype is Ogawa, whereas Hikojima is sporadic, rare, and unstable in the environment [7]. Non-O1 and non-O139 *V. cholerae* strains habitually inhabit rivers and estuarine areas against O1 and O139 strains. Studies have shown that non-toxigenic environmental strains could be switched to toxigenic strains through transduction with cholera toxin (CT)-encoded phage CTXϕ [8].

### 1.2. Vibrio parahaemolyticus

The halophilic pathogenic microorganism *V. parahaemolyticus* is widely distributed worldwide in coastal waters and is commonly found in seafood, sometimes in numbers as high as 10^3^–10^4^/g in oysters and <10^2^/g in tropical shrimp [9]. *V. parahaemolyticus* is free-swimming or attached to organisms such as zooplankton, fish, shellfish, and sediment. Food-borne infections with *V. parahaemolyticus* occur when shellfish such as oysters and clams are consumed raw or minimally cooked. Severe watery diarrhea, abdominal cramps, vomiting, nausea, and fever are some of the symptoms of *V. parahaemolyticus* infection. It can also cause infections in open wounds exposed to contaminated water. However, all *V. parahaemolyticus* are not pathogenic, but those manufacturing either a thermostable direct hemolysin (TDH) factor or a TDH-related hemolysin (TRH) are pathogenic [10]. The TDH and TRH virulence factors are encoded by *tdh* and *trh* genes with about 70% nucleotide sequence similarity [11]. Studies have reported that <1% of *V. parahaemolyticus* seafood isolates are *tdh*^+^, while the incidence of *trh*^+^ *V. parahaemolyticus* is considerably higher, with some investigations reporting as high as 60% [9]. Since the mid-1990s, a pandemic clone of O3:K6, first detected in Calcutta, India, has been responsible for many outbreaks in Asia and the USA [12]. These strains harbor the *tdh* gene but not *trh*. *V. parahaemolyticus* can be isolated from farmed shrimp, and recently, early mortality syndrome (EMS) has been ascribed to *V. parahaemolyticus* in *Litopenaeus vannamei* farms [13].

### 1.3. Vibrio vulnificus

*V. vulnificus* is another important human pathogenic vibrio associated with fish and shellfish. *V. vulnificus* is known to cause serious infections in people with compromised immunity, liver diseases, and iron overloaded conditions, with a mortality rate as high as 30–75% [14]. *V. vulnificus* infections are linked with eating raw molluscan shellfish, which accumulate this pathogen from surrounding waters. *V. vulnificus* is responsible for about 95% of deaths associated with the consumption of seafood. *V. vulnificus* can cause fatal wound infections because the microorganism can enter the circulatory system and cause septicemia. Low salinities (5 to 25 ppt) and warm temperatures (20 to 35 °C) have been reported to be favorable for this organism [15,16]. Most cases of *V. vulnificus* infection are reported from tropical and subtropical regions. Based on their biochemical characteristics, *V. vulnificus* are classified into three biotypes, with Biotype 1 responsible for severe human infection and are found naturally in marine and estuarine waters, and biotype 2 are eel pathogens. Biotype 3 is a known hybrid of biotypes 1 and 2 found in freshwater fish in Israel [14].

## 2. Efflux Pumps and Antibiotic Resistance

The recalcitrant nature of specific toxigenic and non-toxigenic strains of *Vibrio* spp. can be problematic in treating severe cases of clinical infections [17]. In general, bacterial resistance to antimicrobial agents falls into several categories [18]. These microbial mechanisms include the formation of biofilms, which can permit communication between various species and, thus, facilitate the development of persister cells [19]. Biofilm communities harbor multiple microbial species enclosed within polymeric matrix systems. These biofilms are frequently associated with various surfaces and provide protection from antimicrobial agents. In many cases, microorganisms within biofilms undergo communication using quorum-sensing molecules to alter their biochemical properties. Another common mechanism of bacterial resistance involves the enzymatic alteration of drug targets, such as the ribosome or cell wall synthesizing machinery [20]. The altered bacterial targets can exhibit reduced antimicrobial drug binding affinities, preventing the growth-inhibiting properties permitting continued microbial growth. Frequently, target alterations result from spontaneous mutation and insertional mutagenesis by mobile genetic elements. Protection of the antimicrobial drug target prevents the binding to cytoplasmic cell growth machinery [21]. A well-studied target protection system involves the tetracycline ribosomal protection proteins Tet(O) and Tet(M), which are homologous to the elongation factor G (EF-G) of prokaryotic ribosomes. When bound to the 30S ribosomal subunit, Tet(O) and Tet(M) proteins remove or prevent the binding of tetracycline to the A-site of the ribosome, permitting translation to proceed unabated [22]. The enzymatic-based destruction of antimicrobial agents is an important virulence mechanism [23]. These resistance systems hydrolytically render antimicrobial agents into inactive forms. Of particular concern are the extended-spectrum β-lactamases (ESBLs) that cleave the β-lactam functional moieties of cephalosporins, penicillins, and associated β-lactam-harboring drugs. Prevention of antimicrobial permeability to the cytoplasm of bacterial cells represents a well-known drug resistance system [24]. These resistance determinants are known to reduce the expression of antimicrobial entry systems in the bacterial membrane or inactivate such drug entry proteins by mutation. Similarly, the lipopolysaccharide components of the bacterial cell wall can confer impermeability of extracellularly located antimicrobial agents. The energy-dependent efflux of multiple antimicrobial agents from bacterial cells of the *Vibrio* spp. is a widely recognized resistance mechanism [25,26]. These active efflux pumps can exploit the energy stored in ATP in primary active transport systems or the energy stored in ion-based electrochemical gradients in secondary active transporters. Many of these secondary active efflux pumps are called antiporters, which exchange drug and ion during transport and have multiple structurally distinct antimicrobial substrates.

### 2.1. General Mechanisms of Antibiotic Efflux Pumps

Inside bacterial cells of *Vibrio* species where the cellular targets for antimicrobial agents reside, the growth inhibitory effects of such intracellularly located drugs are diluted by various solute drug efflux systems [27]. These solute transport systems actively pump antimicrobial agents to the extracellular milieu, permitting bacteria that harbor these drug efflux pumps to grow and predominate under relatively high concentrations of structurally distinct antimicrobial agents, including those clinically relevant drugs in the chemotherapy of infectious disease [28]. Active transport of antimicrobial agents across the bacterial membranes represents a critical efflux-based mechanism for pathogen resistance to clinically relevant antibacterial agents [29].

In primary active transport systems, the energy stored in ATP is utilized by its hydrolysis to actively accumulate their water-soluble substrates on one side of the membrane [30]. Several species of the *Vibrio* genus harbor these primary active drug pump systems, which are described below. Another active transport system involves the biochemical modification of substrate during transport across the membrane, as exemplified by the phosphoenolpyruvate-dependent phosphotransferase system (PTS), also known as group translocation [31]. While sugar-alcohols and antimicrobial agents are included as substrates for *Vibrio* microorganisms [31,32,33], such active solute transport systems are utilized to enter bacterial cells.

Likewise, *Vibrio* species possess secondary active transport systems for drug efflux, which use the energy stored in the form of ion gradients across the membrane [34]. These ion-based electrochemical gradients, such as those involving sodium or protons, catalyze the accumulation of antimicrobial agents on one side of the membrane during a translocation process called antiport [35,36]. Together, these primary and secondary active transporters in bacteria account for a significant public health concern as mediators of potentially untreatable multidrug-resistant infectious disease-causing bacterial pathogens.

### 2.2. Classification of Antimicrobial Efflux Pumps

Regarding the active extrusion of antimicrobial agents, several large superfamilies of efflux pumps have been characterized in bacteria [26]. Of particular interest is the RND (resistance-nodulation-division) superfamily of drug and multidrug transporters driven by a secondary active transport mode of energization [37]. The major facilitator superfamily (MFS) is another relevant and large superfamily of transporters but is driven by passive and secondary active solute transport systems. The established multidrug and toxic compound extrusion (MATE) superfamily contains several drug efflux systems in various species of Vibrio [38,39]. Transporters of the small multidrug resistance (SMR) superfamily [40] have been classified as progenitors of the large drug metabolite transporter (DMT) superfamily [41]. More recently, transport systems that are homologous to members of the so-called proteobacterial antimicrobial compound efflux (PACE) family, such as AceI, were discovered in *V. parahaemolyticus* [42]. The ATP-binding cassette (ABC) superfamily harbors many members, which use ATP hydrolysis as the main mode of energy for primary active solute transport [43].

### 2.3. Efflux Pumps of RND Family in Vibrio Species

The resistance-nodulation-cell division (RND) superfamily of membrane transporters is composed of a tripartite system with an outer membrane protein (OMP), an inner membrane protein (IMP), and a periplasmic membrane fusion protein (MFP) [44]. Different components of the RND efflux pump are generally encoded on an operon. The proteins work in synergy to extrude a compound outside the cell, and the absence of any single protein of this tripartite system makes it dysfunctional [45]. IMP is the antiport protein energized by the protons (H^+^), while the MFP is an adaptor protein that connects OMP with the IMP [46,47]. As a result, the RND pump is organized into a continuous channel that allows direct exportation of the compounds from the cytoplasm to the exterior without entering the periplasmic space, thus making them a very effective drug resistance mechanism for Gram-negative bacteria [45,47,48,49]. The crystal structures AcrAB-TolC proteins of *Escherichia coli* [50,51] and the MexAB-OprM of *Pseudomonas aeruginosa* [52,53,54] have helped decipher the structure-function relationships in these RND efflux pumps [55]. RND pumps are non-specific and extrude structurally diverse and unrelated substrates across the membrane, although RND efflux pumps’ substrates are characteristically lipophilic [45,55]. Cationic, anionic, and uncharged substances and substances with multiple ionizable groups are efficiently handled by the RND pumps [45,55].

Some of the well-characterized RND efflux pumps from *V. cholerae* O1 include VexLM, VexIJK, VexGH, VexEF, VexCD, as well as VexAB and contribute to bile acid and antimicrobial resistance [56,57,58]. Higher expression of *vexAB* and *vexCD* genes in the presence of bile substantiates the role of these efflux pumps in bile resistance, and this might positively contribute to successful colonization of the small intestine by *V. cholerae* [57,58]. Genome comparison of *V. cholerae* O1 and non-O1 serovars revealed the presence of all but one (VexE) efflux pump in the genome of non-O1 strain PS15 [59]. The TolC OMF is essential for the functioning of most of these efflux pumps. VexAB, VexCD, and VexEF could be expressed in a hypersensitive *E. coli* (∆*acrAB*, ∆*ydhE*, *hsd*^−^, ∆*tolC*) background with the help of the TolC outer membrane factor from *V. cholerae* (TolC_vc_) [60]. Of these, VexAB and VexEF exhibited a broader substrate range that included multiple antibiotics such as erythromycin, novobiocin, and dyes and detergents, while the efflux activity of VexCD was restricted to bile acids and detergents. Apart from bile salts, certain antibiotics such as ampicillin and novobiocin have been reported as substrates for the VexH pump since the deletion of the *vexH* gene in a VexB-deficient *V. cholerae* resulted in higher susceptibility to these antibiotics [58]. VexEF is dependent on the electrochemical gradient of Na^+^ ions, unlike all other RND pumps energized by H^+^, and was the first such RND efflux pump described from bacteria [60].

No antibiotic substrates have been identified so far for VexIJK and VexLM pumps [58,60]. These efflux pumps might participate in other physiological activities essential for the survival and persistence of *V. cholerae* in the host and the environment. It has been shown that VexB, VexD, VexH, and VexK are necessary for intestinal colonization and production of virulence factors such as the cholera toxin (CT) and toxin co-regulated pilus (TCP) [58]. A majority of RND efflux pumps have bile acid as their substrate, and considering the importance of bile in the physiology of enteric bacteria [61], the role of these efflux pumps in the virulence gene regulation has been a topic of significant research interest. Before intestinal colonization, resistance against the toxic effect of the bile salts is accomplished by the ToxR-mediated repression of *ompT*, which has a negative role in bile salt resistance [62]. At the same time, ToxR activates the expression of *ompU*, and the OmpU-producing strains are more resistant to bile [63]. The increased efflux of bile salts follows OmpU production and transport by the RND efflux pumps. A *V. cholerae* mutant strain lacking all RND efflux pumps exhibited increased susceptibility to bile, decreased cholera toxin production, and an inability to colonize infant mouse small intestines [57,64]. Together, this evidence emphasizes the critical role that RND efflux pumps play in antimicrobial resistance and host persistence and virulence of *V. cholerae*.

The genome of *V. vulnificus* has 11 putative RND efflux pump-encoding genes, three of which (homologues of *V. cholerae* VexAB, VexCD, and AcrAB of *E. coli*) have been characterized by gene deletion studies [50]. A study identified the *norM* gene in whole-genome sequences of clinical *V. vulnificus* isolates [65]. While a mutant *V. vulnificus* lacking the *vexAB* homolog was more susceptible to erythromycin, acriflavine, ethidium bromide, and bile acid, deletion of *acrAB* homolog resulted in increased sensitivity of acriflavine alone, and *vexCD* deletion did not have any effect on the susceptibility to any of the antibiotics, dyes or bile acid [66]. Two TolC homologs, TolCV1 and TolCV2 in *V. vulnificus*, have been involved in resistance to antibiotics and inhibitory dyes. *V. vulnificus* mutants lacking TolCV1 and TolCV2 exhibited increased susceptibility to antibiotics novobiocin, erythromycin, and tetracycline, while TolCV1 mutant was also susceptible to DNA intercalating dyes (ethidium bromide, acriflavine) and detergents (bile acids, SDS) and exhibited reduced motility [67]. The expression of the *tolCV1* and *tolCV2* increased when the bacterium was exposed to antibiotics and other chemicals [66]. TolCV1 and TolCV2 could partially complement the TolC protein of *E. coli* by interacting with the AcrA protein of the AcrAB-TolC efflux system of *E. coli* [68]. Although VceC is functionally identical with the TolC and OprM outer membrane factors, they share minimal (<10%) amino acid similarity among them [69,70].

The outer membrane component TolC is generally essential for the function of RND efflux pumps irrespective of bacterial species. In certain instances, however, the TolC components of one species cannot functionally replace the TolC of the other. For example, the MICs of antibiotics for VexEF were much higher with TolC from *V. cholerae* (TolC_vc_) in an *E. coli* background, compared to the MICs of VexEF with TolC of *E. coli* (TolC_EC_) itself, although both share 47% identity and 7% similarity at the amino acid level [60]. Similarly, the RND efflux pumps of *E. coli* could not function with the TolC from *V. parahaemolyticus*, suggesting a species-specific preference for the TolC component [71,72]. TolC in the *V. cholerae* O1 El Tor strain has been reported to play an essential role in the transcription of the ToxR regulon, a finding that emphasizes the importance of efflux pump-mediated regulation of virulence in pathogenic bacteria [73].

### 2.4. MATE Efflux Pumps in Vibrio Species

The norfloxacin efflux pump NorM of *V. parahaemolyticus* was the first Na^+^/drug antiporter to be reported from bacteria with no affiliation to any known transporter protein groups known at its time of discovery [74,75]. NorM is energized by Na^+^ unlike all other known H^+^/drug antiporters and shared high homology with YdhE protein of *E. coli*, as well as similar protein sequences in diverse bacterial groups [74,75]. Thus, NorM and its homologous proteins were placed under a new family of transporter proteins called MATE (multidrug and toxic compound extrusion) [76]. Subsequent studies by this research group identified amino acids Asp32, Glu251, and Asp367 located in the transmembrane region as essential for the Na^+^-dependent drug efflux activity in NorM [77]. However, recent studies have reported on MATE efflux proteins driven by H^+^ electrochemical gradients, such as DinF-BH, NorM-PS, and VcmN [78,79,80]. NorM-VC of *V. cholerae*, by contrast, has been reported to be coupled to both Na^+^ and H^+^ [81]. The MATE family of proteins extrudes diverse compounds, including antibiotics and anticancer drugs, particularly the hydrophobic and weekly cationic molecules. However, despite structural similarities, MATE transporters differ significantly in their efflux mechanisms, substrate profiles, and proton couplers [79,82].

Based on mutational and molecular dynamics simulation studies, amino acid residues Asp36, Glu255, and Asp371 in NorM-Vc are presumed to be important for the substrate binding and transport activity, corresponding to Asp32, Glu251, and Asp367 in NorM from *V. parahaemolyticus* [77,83,84,85]. While Asp36 is critically important for both Na^+^ and H^+^ induced conformational changes in TM1, Thr200 is essential for Na^+^-mediated conformational transition alone [85,86].

VcmA is a Na^+^/drug antiporter of the MATE family in *V. cholerae* non-O1 and is capable of effluxing multiple antibiotics, including hydrophilic quinolones (norfloxacin, ciprofloxacin, ofloxacin) and aminoglycosides (streptomycin and kanamycin), but not hydrophobic quinolones such as sparfloxacin and nalidixic acid [87]. This 457 amino acid long efflux protein with 12 putative transmembrane domains showed high sequence homologies with NorM of *V. parahaemolyticus* and YdhE of *E. coli* [87]. The presence of VcmA has been found in the whole genome sequence of both *V. cholerae* O1 and non-O1 [59,88]. Another Na^+^/drug antiporter-type multidrug efflux pump VcrM has been reported from *Vibrio cholerae* non-O1 [39]. This 445 amino acid containing pump folds into 12 TMS and belongs to the DinF-subfamily within the MATE family of transport proteins. The efflux of several chemicals and dyes was dependent on Na^+^ or Li^+^ [39]. However, no antibiotic substrate has been identified for VcrM so far.

**Figure 1:**
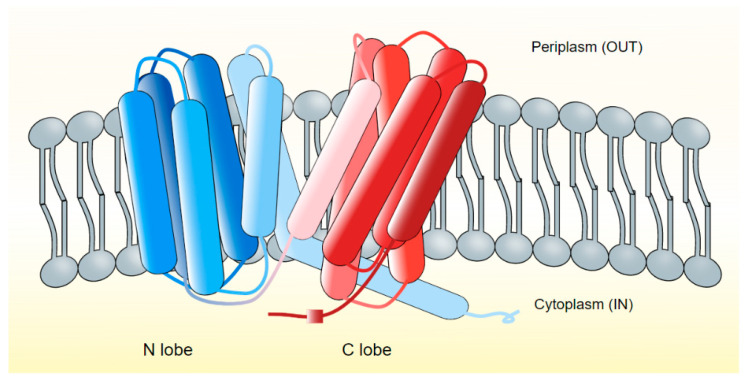
MATE transporters of bacteria. The transporters are grouped under NorM and DinF subfamilies and are generally composed of 12 TM helices which fold into two distinct N- (TM1-TM6) and C- (TM7-TM12) lobes [76,84,93]. The topology of MATE transporters is characteristically distinct from that of MFS transporters, many of which have similar 12 TM helices [76,94]. In the NorM subfamily (NorM-VC), the C-lobe functions as a cation binding site, and two acidic amino acid residues, Glu255 and Asp371, are located between TM7-12, are critically important for this function [83].

The X-ray crystallographic structures of H^+^-dependent MATE efflux protein PfMATE from *Pyroccocus furiosus* [89], VcmN from *V. cholerae* [79], ClbM of *E. coli* [90], and the Na^+^-dependent NorM-Vc from *V. cholerae* [83], and NorM-NG from *Neisseria gonorrhoeae* [91] have been elucidated. NorM with 12 transmembrane helices folds into two bundles of 6 helices each, termed C lobe and N lobe, placed perpendicular to the membrane [92] (Figure 1).

**Figure 2:**
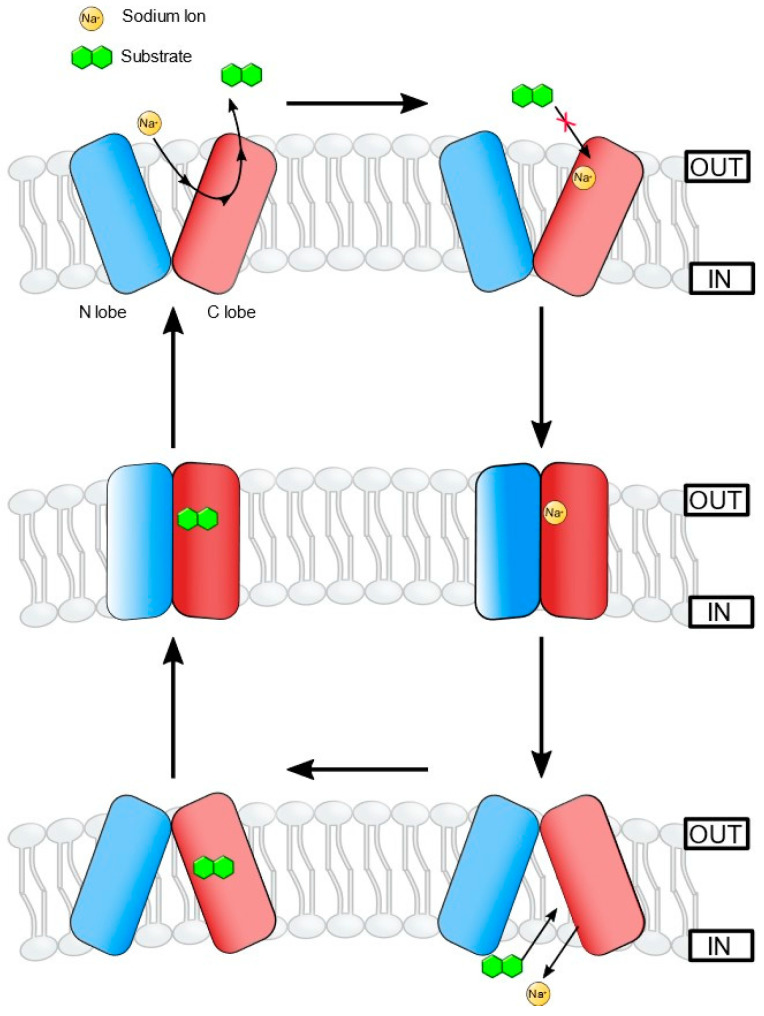
Based on the crystal structures of PfMATE (H^+^-coupled DinF protein from *Pyrococcus furiosus* [89,98], Kusakizako and colleagues have proposed an “alternating access mechanism” for the DinF-subfamily of MATE transporters [83]. Binding with Na^+^ (or H^+^) ions occurs at the Asp residue in the TM1 (N-lobe), which results in the bent conformation of TM1. In this state, TM1 assumes an outward open state. No substrate-binding takes place due to changes in the conformation at this stage. The following proposed form is a cation-bound occluded state, followed by an inward-open bent form, assuming a straight conformation to allow substrate binding. This step is followed by a substrate-bound occluded state, which finally enters into outward-open conformation.

The MATE transporters adopt an “alternating-access model”, switching between the substrate-bound outward-facing (OF) or ion-bound inward-facing (IF) conformations, which allows binding of either the substrate or the ion (Na^+^) but not both, in a single conformation state [84,89,95] (Figure 2). The C and N lobes with bundles of six helices each form the OF conformation with a V-shaped central cavity facing the extracellular environment [96]. In this conformation, the central cavity is accessible to the substrate from the extracellular side only. Using molecular dynamics (MD) simulations and double electron-electron resonance (DEER) spectroscopy, Castellano and colleagues identified a Na^+^ binding site in the N-terminal lobe, which induces conformational changes in the protein upon binding with Na^+^ [39,86,97]. Crystal structures of VcmN in two different pH-associated conformations showed protonation-induced conformational changes in TM1 due to the rearrangement of hydrogen bonds in the N-lobe [79]. The polar amino acid Asp35 is critically important for this process, as a mutation in Asp35 abolished proton and ethidium bromide transport activities. The two different conformations determine the binding of H^+^ or the substrate, and not both simultaneously, and the associated rearrangement of hydrogen bonds around Asp35 disallows re-binding of the substrate after extrusion [79].

The observed antimicrobial extrusion activities of efflux pumps in the laboratory might not represent the more prominent role of efflux pumps in the survival and persistence of bacteria in the environment. For example, the plant pathogen *Erwinia amylovora* employs a NorM efflux pump to overcome the inhibitory activities of compounds produced by co-inhabiting epiphytic bacteria and proliferate to numbers sufficient to cause successful infection of the plants [99].

### 2.5. Efflux Pumps of MFS Family in Vibrio Species

The first MFS antimicrobial transporter with multiple substrates discovered in *V. cholerae* was VceB [100]. This efflux pump was predicted to have 14-transmembrane (TM) domains and contain in its fifth TM the hallmark “antiporter motif”, also known as Motif C [100,101]. Interestingly, VceB forms a part of a more extensive multi-component transport system, VceCAB [102], controlled by VceR, a transcriptional regulator [103]. The VceA protein constitutes a periplasmic component called a membrane fusion protein, and the VceC component plays a role as an outer membrane channel protein [70,102]. Together, these elements assemble into a so-called tripartite multidrug efflux system for the export of multiple structurally different antimicrobial agents from cells of *V. cholerae* [102]. More recently, the VceAB two-component translocase was discovered to participate with an outer membrane protein from *Pseudomonas aeruginosa*, OprM, in the assembly of another tripartite system, VceAB-OprM, to export multiple antimicrobial agents [104].

Our laboratory discovered that the genetic determinant, denoted as *emrD-3*, from a *V. cholerae* O395 genome project, encoded an MFS multidrug efflux pump that conferred resistance to multiple structurally distinct antimicrobial agents and actively exported ethidium bromide in an energy-dependent manner [105]. Interestingly, a genome comparison study showed that EmrD-3 is encoded on chromosome II of a toxigenic *V. cholerae* strain N16961 but is missing in a non-toxigenic environmental Puget Sound isolate *V. cholerae* strain PS15 [59,88,106]. The EmrD-3 multidrug efflux pump was predicted to harbor 12-TM and the antiporter motif [71,107]. Multiple sequence comparisons showed a high degree of relatedness to various putative exporters from several unrelated bacterial species, such as *Bacillus cereus*, *Proteus mirabilis*, *Shewanella putrefaciens*, plus taxonomically related microorganisms *V. vulnificus*, *V. parahaemolyticus*, and *V. harveyi* while containing the antiporter motif [105]. To our knowledge, however, these and other putative MFS homologs have not been characterized physiologically.

In *V. parahaemolyticus*, the *pvsC* gene encodes an MFS protein and is a component of the *pvsABCDE* operon for metabolism and transport of ferric vibrioferrin [108,109]. The PvsC substrate vibrioferrin is an iron siderophore and confers the export of vibrioferrin from the bacterium [108,109]. The PvsC transporter thus appears to function in siderophore expulsion, a suggested protective mechanism, an established virulence factor for bacterial pathogens [110].

More recently, the five determinants denoted *mfs1-5* from an El Tor *V. cholerae* strain encode MFS proteins that, when mutated by deletion, lose the ability to confer resistance to crude bile and tetracycline [111]. Interestingly, the expression of these *mfs* genes (*mfs1-5*) appeared to be under the control of a transcriptional regulator called MfsR [111], a homolog of the well-known LysR protein, and responsive to tetracycline as an inducer among other unidentified putative inducers in extracts of mouse intestinal tissue [111,112].

In 2017, our laboratory discovered that garlic extract from *Allium sativum* and its bioactive agent allyl sulfide inhibited the antimicrobial efflux activity of EmrD-3 from *V. cholerae* [113], indicating that the EmrD-3 multidrug efflux pump is a good target for resistance modulation and inhibition of growth for the cholera pathogen [59,114,115]. Furthermore, we showed that elements of *A. sativum* extract exhibited profound synergy when combined with clinically critical antimicrobial agents, like lincomycin, vancomycin, and others [113]. These synergistic studies predict that EmrD-3 participates as part of an overall tripartite efflux system, such as that seen for MFS pumps of *Vibrio* species. Lastly, it remains to be determined whether allyl sulfide itself is another substrate or gene expression regulator of the EmrD-3 multidrug efflux pump.

**Figure 3:**
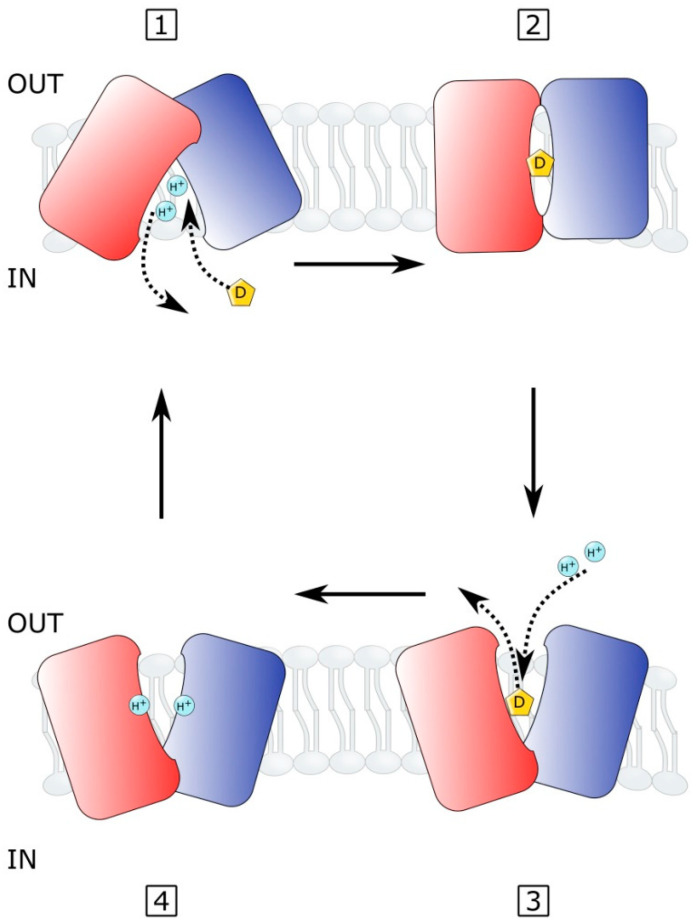
A rocker-switch mechanism was proposed for MFS transporters. In this proposed antiport system, proton-driven drug efflux occurs through the pump by alternately exposing the drug (D) binding site to either side of the membrane. The drug translocation process involves conformation changes in the two bundles or halves of the MFS pump to form inward open, occluded, and outward-facing open conformations (steps 1–4 in the figure) [118,119].

The multidrug efflux systems of the MFS are known to share similar amino acid sequences and protein structures, predicting that the MFS transporters share a common mechanism of solute transport across the membrane [101,116,117]. In the so-called rocker-switch alternating-access mechanism of solute transport, the antiport of drug versus ion involves conformational changes that expose the drug-binding site to inward versus outward-facing versions [27,118,119] (Figure 3). In this rocker-switch mechanism, a single antimicrobial substrate-binding site is proposed for MFS efflux pumps in which the transporters alternate access to the drug-binding site between one or the other side of the membrane [120]. The mechanism involves the following process [119] (Figure 3). (1) The inward-facing transporter undergoes proton release, permitting the antimicrobial drug to bind the inward-facing drug-binding site. (2) The antimicrobial pump is bound to drug and deprotonated, destabilizing the inward-facing structure to form an occluded state. (3) A conformational change in the pump follows in which the drug-binding site orients to an outward-facing conformation, releasing the drug to the other side of the membrane. The proton motive force permits the protonation of the outward-facing empty pump. (4) The protonated pump induces another conformation change to reorient the drug-binding site to an inward-facing conformation, thus allowing intracellularly located drug to bind the pump, allowing deprotonation in which the released protons enter the cell, completing the drug/H^+^ antiport process [119].

A detailed postulated catalytic mechanism for multidrug efflux pump systems is thought to involve several steps, as predicted by kinetic studies of secondary active drug transport [121,122]. First, (a) an extracellular proton binds to an outward-facing empty pump; (b) the affinity of the inward-facing binding site for the drug is enhanced on the pump; (c) the inward-facing pump binds the drug. (d) A conformation change exposes the antimicrobial to the outward-facing state and the proton to the inward face. (e) The drug is released outside; (f) the proton is released inside the cell; lastly, (g) the emptied pump reorients so that the substrate-binding site faces inward, and the proton-binding site faces outward. The emptied multidrug efflux pump is ready to undergo the drug transport cycle again [36,121,122]. In both of these transport models, it is unknown how the energy stored in the ion-motive force is transduced to drive the drug translocation across the membrane, especially in cases where multiple structurally diverse antimicrobial agents actively accumulate their substrates on one side of the membrane.

In the MFS antimicrobial exporters, the highly conserved amino acid sequence motif C (antiporter motif) has been shown to play a molecular hinge role during the conformational alterations in drug antiport across the membrane [123,124]. Furthermore, as predicted in earlier studies [101,116], the α-helical structure of the fifth TM of MFS transporters is kinked, as demonstrated by various crystal structure studies [27,125,126]. A recent study reported that the TM structure formed by the antiporter motif twists about itself during drug efflux and plays a role in orienting the empty transporter during the catalytic transport cycle [127]. The critical nature of the antiporter motif structure in MFS efflux pumps predicts that the hinge structure formed is a good target for efflux pump inhibition and consequent restoration of effective chemotherapy towards cholera [123,128].

### 2.6. Efflux Pumps of ABC Superfamily in Vibrio Species

The ABC (ATP-binding cassette) transporters derive energy from the hydrolysis of ATP to move diverse compounds such as sugars, amino acids, antibiotics, anticancer drugs, peptides, etc., across the membranes and are widely distributed among both eukaryotes and prokaryotes [129]. A typical ABC pump consists of two membrane-integral part domains that transverse the membrane six times each (12 TMS in total) and two ATP hydrolyzing domains [130]. In many Gram-negative bacteria, ABC transporters are an integral part of toxin secretion systems and play crucial roles in their virulence. *V. cholerae* genome has several ABC transporters predicted to be involved in the transport of amino acids, vitamins, peptides, sugars, lantibiotics, etc. [59]. VcaM was the first ABC transporter functionally characterized from *V. cholerae* [89]. This efflux pump confers resistance to ciprofloxacin, norfloxacin, tetracycline, doxorubicin, daunomycin, and dyes such as 4′,6-diamidino-2-phenylindole (DAPI), and Hoechst 33342, and the efflux activity was inhibited by reserpine and sodium o-vanadate [39]. More recently, evidence suggests that VcaM relies on the outer-membrane component TolC for active extrusion from host cells [131].

## 3. Efflux Pumps and Virulence

The RND efflux system in *V. cholerae* coded by six operons: *vexAB*, *vexCD*, *vexEF*, *vexGH*, *vexIJK*, and *vexLM* operate with the same chromosomal element, *tolC*, encoding an outer membrane protein. These efflux pump systems have significant roles to play in the *V. cholerae* infections by colonizing the small intestine [3]. This colonization could be done by imparting resistance to inhibitory substances like bile salts and organic acids that hamper bacterial growth in the intestinal habitat [64,132,133]. The expression of VexD is induced in the presence of bile salts [132]. The expression of the important virulence factors of *V. cholerae*, such as cholera toxin (CT) and the toxin-coregulated pilus (TCP), is regulated by RND efflux pump systems. The *toxT* gene encodes a transcriptional activator responsible for transcription of the genes encoding for CT. The efflux systems VexM and VexF are also implicated in supporting the expression of virulence factors [58]. The mechanism by which RND efflux systems regulate the virulence factor expression is still not explained in detail. Efflux pump inhibitors can inhibit the expression of tcpPH and toxT genes as well as the production of CT and TCP, suggesting a complex interaction between virulence factors and the RND efflux pumps [134]. The evidence also suggests that the effect of efflux pump inhibitors on virulence genes could involve mechanisms other than the inhibition of efflux pumps alone [134]. The outer membrane protein TolC, which is part of the tripartite efflux system, participates in the secretion of RtxA1 toxin in *V. vulnificus* [135].

Nevertheless, it has been proposed that removing redundant function systems results in the accumulation of a low molecular weight compound. This agent acts as a negative effector molecule functioning as a regulator of the expression of the important virulence factors. The life cycle of *V. cholerae* associated with zooplankton and other sediment-dwelling organisms in the aquatic environment could also be correlated with the significance of a few RND efflux systems that have no role in other resistance mechanisms [3].

### Role in Quorum Sensing and Biofilm Formation

Biofilm is an aggregation of surface-attached bacterial cells associated with biotic and abiotic surfaces embedded in an extracellular polymeric matrix [136]. The bacterial composition of biofilms can vary, from a single species to multiple species, and the biofilms have a multilayered architecture [137]. Bacteria in biofilms have physiology distinctly different from their planktonic counterparts, being more resistant to antibiotics and disinfectants as well as changes in physicochemical conditions of the surrounding environment [138]. Low molecular weight extracellular compounds like homoserine lactones are produced by bacterial cells of *Vibrio* sp. in biofilm for intercellular communication. Quorum sensing is the prominent mechanism by which bacteria regulate physiological activities by detecting and responding to the levels of these extracellular compounds. Bacteria produce biofilm once the bacterial cells reach a specific density as detected by quorum sensing, which also regulates the expression of virulence factors [139]. Multidrug-resistant membrane efflux pumps in *Vibrio* sp. could be associated with the expression of virulence factors regulated by the quorum-sensing mechanism. The compounds involved in the bacterial quorum-sensing mechanism require membrane transporters similar to efflux pumps. Hence, the inhibition of efflux pumps could reduce biofilm production and other virulence factors [140].

## 4. Conclusions

Membrane efflux pumps play a critical role in the survival physiology of marine *Vibrio* species by contributing to the bacterial ability to survive antimicrobial substances and high osmolarity, biofilm formation, quorum sensing, and even virulence. Although the whole genome sequences have allowed the identification of new efflux pumps in the genome of *Vibrio* bacteria, some of which do not contribute to resistance to any known antimicrobials, their exact functions have remained elusive. Crystallographic structures of some efflux proteins of *Vibrio* species and other closely related bacteria have allowed an understanding of the structure-function relationships of these efflux pumps to a greater extent. Much remains to be understood regarding the roles of efflux pumps in the natural environment of *Vibrio* species, their substrate profiles, regulation, and transport mechanisms.

## Data Availability

Not applicable.
